# Brain Metabolism Changes in Patients Infected with HTLV-1

**DOI:** 10.3389/fnmol.2017.00052

**Published:** 2017-02-28

**Authors:** Manuel Schütze, Luiz C. F. Romanelli, Daniela V. Rosa, Anna B. F. Carneiro-Proietti, Rodrigo Nicolato, Marco A. Romano-Silva, Michael Brammer, Débora M. de Miranda

**Affiliations:** ^1^Faculdade de Medicina, Instituto Nacional de Ciência e Tecnologia de Medicina Molecular, Universidade Federal de Minas GeraisBelo Horizonte, Brazil; ^2^Fundação HemominasBelo Horizonte, Brazil; ^3^Grupo Interdisciplinar de Pesquisas em HTLV – GIPHBelo Horizonte, Brazil; ^4^Departamento de Saúde Mental, Faculdade de Medicina, Universidade Federal de Minas GeraisBelo Horizonte, Brazil; ^5^Department of Neuroimaging, Institute of Psychiatry (PO89)London, UK; ^6^Departamento de Pediatria, Faculdade de Medicina, Universidade Federal de Minas GeraisBelo Horizonte, Brazil

**Keywords:** Gaussian processes, positron emission tomography, 18f-fluorodeoxyglucose, tropical spastic paraparesis, HTLV-1, HTLV-associated myelopathy

## Abstract

The Human T-cell leukemia virus type-I (HTLV-1) is the causal agent of HTLV-associated myelopathy/Tropical Spastic Paraparesis (HAM/TSP). HAM/TSP is the result of demyelination and cell death in the spinal cord and disruption of the blood-brain barrier (BBB), mediated by a virus-induced inflammatory response. In this study, we applied Positron Emission Tomography with 18F-fluordeoxyglucose (18F-FDG PET) to evaluate brain metabolism in a group of 47 patients infected with HTLV-1, and 18 healthy controls. Patients were divided into three groups according to their neurological symptoms. A machine learning (ML) based Gaussian Processes classification algorithm (GPC) was applied to classify between patient groups and controls and also to organize the three patient groups, based on gray and white matter brain metabolism. We found that GPC was able to differentiate the HAM/TSP group from controls with 85% accuracy (*p* = 0.003) and the asymptomatic seropositive patients from controls with 85.7% accuracy (*p* = 0.001). The weight map suggests diffuse cortical hypometabolism in both patient groups when compared to controls. We also found that the GPC could separate the asymptomatic HTLV-1 patients from the HAM/TSP patients, but with a lower accuracy (72.7%, *p* = 0.026). The weight map suggests a diffuse pattern of lower metabolism in the asymptomatic group when compared to the HAM/TSP group. These results are compatible with distinctive patterns of glucose uptake into the brain of HTLV-1 patients, including those without neurological symptoms, which differentiate them from controls. Furthermore, our results might unveil surprising aspects of the pathophysiology of HAM/TSP and related diseases, as well as new therapeutic strategies.

## Introduction

The Human T-cell leukemia virus type-I (HTLV-1) has been identified as the causal agent of Adult T-cell leukemia (ATL; Yoshida et al., 1982) and HTLV-associated myelopathy/Tropical Spastic Paraparesis (HAM/TSP; Osame et al., 1986). It has also been associated with arthritis, uveitis, dermatitis and polymyositis (Murphy et al., 1989; Yamaguchi and Takatsuki, 1993; Proietti et al., 2005), but its exact role in these disorders is still not clear. HTLV-1 can be transmitted sexually or by blood, blood products, and breast milk, inducing a lifelong chronic infection. HAM/TSP, the most severe neurological expression of the HTLV-1 infection, is a chronic demyelinating disease that affects 2%–3% of seropositive patients, more often women than men and usually beginning in adulthood (Osame et al., 1987; Gessain and Gout, 1992). The disease is characterized by progressive weakness and spasticity of the extremities, hyperreflexia and mild peripheral sensory loss (Osame et al., 1987; Gessain and Gout, 1992; Hollsberg and Hafler, 1993).

Neuropathological findings in HAM/TSP include degradation of white matter within the lateral funiculus of the spinal cord, mainly concentrated in the thoracic and lumbar segments (Levin and Jacobson, 1997). Long-standing lesions in the spinal cord show myelin and axons being replaced by glial scars (Zaninovic, 1999). A study by Morgan et al. (2007) shows similar frequencies of brain white matter lesions in patients with HAM/TSP and asymptomatic HTLV-1 carriers without a clear correlation with the inflammatory status.

Many factors influence the development and progression of HAM/TSP. HTLV-1-specific antibodies and infiltrating T lymphocytes in the peripheral blood and cerebrospinal fluid (CSF), accompanied by the release of pro-inflammatory cytokines, such as tumor necrosis factor-α (TNF-α) and interferon-γ (IFN-γ), are typically observed in symptomatic patients (Kannagi et al., 1992; Umehara et al., 1993; Nagai et al., 2001; Yamano et al., 2002). This inflammatory response seems to be involved in the demyelination and cell death that occurs in the spinal cord. Moreover, it also compromises the integrity of the blood-brain barrier (BBB), making lymphocyte trafficking into the CNS more likely. The integrity of the BBB can be further disrupted by the direct infection of resident CNS cells like astrocytes, as suggested by *in situ* hybridization studies that have localized HTLV-1 Tax RNA in these cells (Lepoutre et al., 2009). Most of the insights gained into the viral CNS cellular infection have been the result of experiments using *in vitro* infection of individual cell populations or *in situ* hybridization of CNS tissue (Lepoutre et al., 2009). Although *in vitro* experiments can contribute with insights, they are inherently physiologically limited.

Positron Emission Tomography with 18F-fluordeoxyglucose (18F-FDG PET) measures glucose metabolism, which is an indirect indication of neuronal and synaptic activity (Sokoloff, 1981; Attwell and Iadecola, 2002) and has the potential to provide new information regarding the *in vivo* effect of the HTLV-1 in the human brain. Little is known about brain metabolic changes in HAM/TSP patients. Taniguchi et al. (2015) have reported hypometabolism in bilateral watershed areas of the middle and posterior cerebral arteries, but their study was based on regions of interest (ROI) and focused only on these areas.

Considering the pathogenesis of the HTLV-1 infection and its effects on the CNS, we hypothesized that patients infected with HTLV-1 would have a distinctive pattern of brain metabolism when compared to healthy controls. Furthermore, we hypothesized that the metabolic activity of the brain could be related to the severity of symptoms and therefore be different between asymptomatic and HAM/TSP patients. Thus, in this study we aimed to characterize brain metabolism in HTLV-1 patients.

## Materials and Methods

### Subjects and Controls

In this study, 47 individuals infected with HTLV-1 (17 males and 30 females, the mean age of 50.8, SD = 11.1 years) were evaluated. Subjects were recruited from the GIPH cohort (1997–2015). This is an open cohort formed mainly by patients infected with either HTLV-1 or HTLV-2 during screening for blood donation at the Hemominas Foundation, in Belo Horizonte, Brazil. Currently, around 400 subjects are being followed by the cohort. Our sample of 47 HTLV-1 infected subjects selected as a convenience sample, representative of the whole cohort, with a mean follow up of 9.74 years (SD = 5.08). All enrolled subjects gave written informed consent to participate in this study and be subjected to clinical examination and a 18F-FDG Positron Emission Tomography scan, and all procedures were approved by the local ethics committees (Conselho de Etica em Pesquisa da UFMG (COEP-UFMG) and Conselho de Etica em Pesquisa da Hemominas (CEP-Hemominas)). Exclusion criteria: concomitant infection with other neurotropic viruses (e.g., HIV), syphilis, hepatitis B and C, Chagas disease, neurologic disease due to other identified causes (stroke, neurocysticercosis, brain trauma, neurodevelopmental delay, etc.,) or less than 4 years of formal education. Also, 18 controls, selected to match the sex and age of the HTLV-1 patients, were chosen from our database of healthy volunteers that were submitted to 18F-FDG PET/CT. Control subjects included only if they had no history or current evidence of psychosis, autism, brain disorders or any genetic or medical disorder associated with cognitive impairment. None of the controls reported the previous or current use of psychotropic drugs. All controls gave written informed consent and allowed their brain image to be used in scientific research.

### Clinical Characterization

Patients were classified according to the Expanded Disability Status Scale (EDSS; Kurtzke, 1983; Weinshenker et al., 1989). EDSS is a method of quantifying neurological disability and monitoring changes in the level of disability over time. The EDSS scale ranges from 0 to 10 in 0.5 unit increments that represent higher levels of disability. Due to high variation in clinical symptoms, HTLV-1 patients were segmented into three different groups: (1) EDSS = 0; (2) EDSS = 1–2; and (3) EDSS = 2.5–10.

### Image Acquisition

Resting-state 18F-FDG PET/CT brain images for patients and controls were acquired in a GE Discovery 690 (GE Healthcare, Millwalke, WI, USA) PET/CT scanner as part of a whole-body scan. Subjects had at least 6 h of fasting before the exam. After an intravenous bolus injection of 5.18 MBq/kg of 18F-FDG, subjects rested for 50 min in a quiet and dark room with minimum stimuli. PET brain images were acquired subsequently, with an acquisition time of 10 min, and reconstructed in a 192 × 192 × 47 matrix using the OSEM (Ordered Subsets Expectation Maximization) algorithm, with two iterations and 20 subsets. Attenuation correction was performed using the CT image.

### Image Processing

Before analysis, each PET image was spatially processed using the Statistical Parametric Mapping toolbox (SPM8, Wellcome Trust Centre for Neuroimaging, 2008) implemented in Matlab 7.12.0 (MathWorks, Natick, MA, USA). This involved gross manual reorientation and approximate definition of the image center point, spatial normalization onto a custom 18F-FDG PET template in MNI space. Smoothing by a 12 mm FWHM Gaussian kernel and scaling of each voxel value by the global mean were done to account for differences in global signal between subjects (Friston et al., 1990). The resulting image was a 91 × 109 × 91 matrix, with 2 mm voxels. A mask was created using SPM’s segmentation algorithm to select only gray and white matter voxels. For this, the *a priori* gray and white matter density image were generated for each subject using SPM’s template, the images from all subjects were combined, and only voxels with a probability greater than 50% of representing gray and white matter were selected. Thus resulted in the sampling of 203590 voxels. The information extracted from each 18F-FDG PET scan is a single 203590 × 1 data vector that “summarizes” each patient gray and white matter metabolism.

### Data Analysis

For the image analysis, we chose a machine learning (ML) based approach. In general terms, ML works by creating a model based on a group of matched input-output pairs (i.e., “learning” from data) and then using this model to predict the output for new “unseen” inputs. It is especially useful when dealing with a high number of predictor variables, like the tens of thousands of voxels in a PET image, associated with a much lower number of samples. The primary outcome measure of ML is how well the model generalizes to new data. This is evaluated trough cross-validation, where the model is iteratively trained on a subset of the data and then tested on the remaining data. That reduces overfitting, which occurs when the algorithm starts to “memorize” training data rather than “learn” to generalize from a trend (Dietterich, 1995). ML is a multivariate approach at the single subject level, which means that it takes into account the distributed pattern of effects across the whole brain. That makes sense as the brain is intrinsically multivariate and the spatial distribution of metabolic measures for gray and white matters are correlated and present many complex interactions between them (Doyle et al., 2015). The method differs from the Statistical Parametric Mapping approach, which is a mass-univariate approach and therefore looks at each voxel separately (Friston, 2007).

Many ML algorithms are available for brain image analysis (Lemm et al., 2011). We opted to use the Gaussian Processes Classification (GPC; Rasmussen and Williams, 2006), because is an elegant and flexible approach for the prediction of binary variables and offers the option to automatic tuning of the kernel parameters from the training data via type-II maximum likelihood. Furthermore, it provides a fully probabilistic prediction, which differentiates it from other ML methods like SVM or LDA, and is of interest in the setting of clinical classification and a small number of samples. GP models have been successfully applied to neuroimaging, providing prediction of symptom severity, pain states, cognitive and disease states (Marquand et al., 2010; Hahn et al., 2011; Mourão-Miranda et al., 2012; Pyka et al., 2013; Young et al., 2013). The method also enables the generation of weight maps showing the most relevant features for classification. The importance of discriminative features to each classification was calculated using the analytical method proposed by Gaonkar and Davatzikos (2013), with a threshold of 0.05.

The GPC implementation available within the kernlab R library (Williams and Barber, 1998; Karatzoglou et al., 2004; R Development Core Team., 2013) was used in this study. The kernel function was set to polynomial, and the initial noise variance and tolerance of termination criteria were both set to 0.001.

## Results

### Subject Groups

The 47 HTLV-1 seropositive patients were divided into three groups according to their EDSS. Group 1 (*n* = 22) consisted of asymptomatic carriers, that is, subjects presenting no clinical sign of neurological deficits as measured by the EDSS scale. Group 2 (*n* = 11) was more heterogeneous than the other two groups, with some subjects showing initial and unspecific symptoms. Group 3 (*n* = 14) consisted of patients with confirmed HAM/TSP. The control group consisted of 18 subjects. Group characteristics are summarized in Table 1.

**Table 1:** **Characterization of Human T-cell leukemia virus type-I (HTLV-1) patient groups and controls**.

Group	EDSS	Mean EDSS	Mean sympt. durat. (years)	Mean follow-up (years)	Subj	Sex	Mean age (years)
Group 1	0	0	0	11 (SD = 4.6)	22	12M/10F	50.1 (SD = 10.4)
Group 2	1–2	1.59	3.2 (SD = 3.1)	9.2 (SD = 6.2)	11	2M/9F	47.8 (SD = 12.6)
Group 3	2.5–10	5.28	13.4 (SD = 9.8)	9.1 (SD = 4.5)	14	3M/11F	54.1 (SD = 10.8)
Controls	–	–	–	–	18	9M/9F	42.0 (SD = 12.5)

*Legend: EDSS = Expanded Disability Status Scale, mean sympt. durat. = mean symptom duration, Subj = subjects, M = male, F = female, SD = Standard deviation*.

### GPC Can Accurately Classify Patients and Controls

We first compared 18F-FDG PET brain images of gray and white matter of each patient group with the corresponding images from controls. For each comparison, we performed leave-one-out cross-validation (LOOCV). This involved removing one subject from each cluster and training the GPC algorithm on the remaining data. The obtained model was then used to predict the class of the two removed subjects (control or patient). This validation was repeated *n* times, where *n* is the number of subjects in each group. The results were put in a contingency table, and the sensibility, specificity and accuracy were calculated. The significance was calculated by repeating the cross-validation a 1000 times after randomly permuting the input labels. We expected that if the GPC algorithm was able to accurately classify subjects according to their group, that would suggest that there is a distinct pattern of metabolic activity across white and gray matter that differentiates the groups.

As data from both groups was used to train the algorithm, each group had to have the same number of subjects arranged into matching pairs. Otherwise, the algorithm would train more on one group, resulting in a biased prediction. Pairs were matched as closely as possible for sex and age (Chen et al., 2007; Kim et al., 2009; Hsieh et al., 2012) so that not all controls were selected for each comparison with the clinical groups. Table 2 contains the results for the GPC (prediction accuracy) together with the number of subjects in each group and the sex and age distributions.

**Table 2:** **Results for Gaussian processes classification of HTLV-1 patient groups and controls**.

Class 1	NS	Sex	Age (SD)	Class 2	NS	Sex	Age (SD)	Acc.	*p*-value
Controls	14	9M/5F	46.4 (10.5)	Group 1	14	9M/5F	46.7 (9.2)	85.7%	0.001
Controls	10	5M/5F	42.9 (12.3)	Group 2	10	2M/8F	45 (12.1)	75%	0.024
Controls	10	6M/4F	49.4 (11.2)	Group 3	10	3M/7F	50.1 (10.1)	85%	0.003

*Legend: NS = number of subjects, M = male, F = female, SD = Standard deviation, Acc = accuracy for GPC. Group 1 = EDSS 0, Group 2 = EDSS 1–2 and Group 3 = EDSS 2.5–10*.

**Figure 1:**
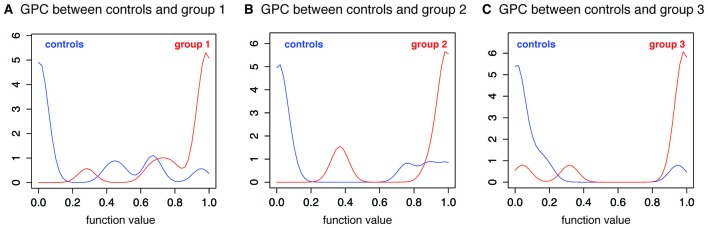
**Gaussian process classification between controls and patient groups.** Histograms for the output of the Gaussian process function. Output values of less than 0.5 represent probability of belonging to class 1 (blue line) and values higher than 0.5 represent probability of belonging to class 2 (red line). **(A)** GPC between controls and group 1 (Expanded Disability Status Scale - EDSS 0). **(B)** GPC between controls and group 2 (EDSS 1–2). **(C)** GPC between controls and group 3 (EDSS 2.5–10).

Classification between controls and the asymptomatic group 1 showed the highest prediction accuracy (85.7%, *p* = 0.001). All patients, except one, were correctly classified as patients, and all, except three control subjects, were properly classified as controls (Figure 1A). The figure depicts the output of the GP function (class probability) and shows that most of the correctly classified subjects had a high probability of belonging to their class. Classification between group 2 and controls was less accurate (75%), but still significant (*p* = 0.024; Figure 1B). Classification between group 3 (HAM/TSP) and controls was more accurate (85%, *p* = 0.003), with one misclassified control and two misclassified patients (Figure 1C).

Since both asymptomatic and HAM/TSP patients could be differentiated from controls with high accuracy, we performed a multiclass classification between controls and these two patient groups. The mean accuracy for the classification was 69.7% (*p* = 0.001), with 81.8% correct classification of controls, 72.7% of group 1 (asymptomatic) and 54.5% of group 3 (HAM/TSP).

### Weight Maps Suggest Diffuse Hypometabolism in Patient Groups 1 and 3

**Figure 2:**
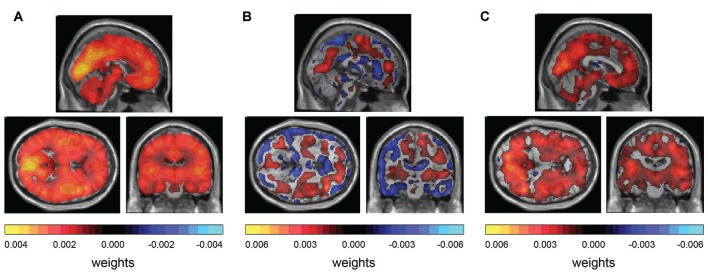
**Weight maps for classification. (A)** GPC between controls and group 1 (EDSS 0) shows diffuse distribution of positive weights. **(B)** GPC between controls and group 2 (EDSS 1–2) shows diffuse distribution of positive weights and negative weights. **(C)** GPC between controls and group 3 (EDSS 2.5–10) shows diffuse distribution of positive weights and negative weights.

To access the contribution of each voxel for the class prediction, we produced weight maps and superposed them on a MRI template in MNI space. The magnitude of the weight is related to the importance of a particular voxel for the prediction and the sign of the weight is related to relative differences between classes: positive weights suggest a tendency for higher values in class 1 and negative weights suggest a tendency for higher values in class 2. The significance of these weights was calculated and only those with a significance of less than 0.05 were used to produce the map. For the classification between controls and group 1, the weight map exhibited a global and diffuse distribution of positive weights, suggesting a diffuse hypometabolism in the patient group 1 (HTLV+ asymptomatic; Figure 2A). For the classification between controls and the more heterogeneous patient group 2, the weight map showed areas of positive and negative influences, suggesting both increased and decreased metabolism in the patients of group 2 when compared to controls (Figure 2B). The pattern of metabolism has a tendency towards a symmetrical distribution when comparing both hemispheres. Finally, for the comparison between controls and patient group 3, the weight map consisted of predominantly positive weights, over again suggesting a diffuse hypometabolism of the HAM/TSP group compared to controls (Figure 2C).

### GPC Can Accurately Classify Asymptomatic HTVL+ and HAM/TSP Patients

**Figure 3:**
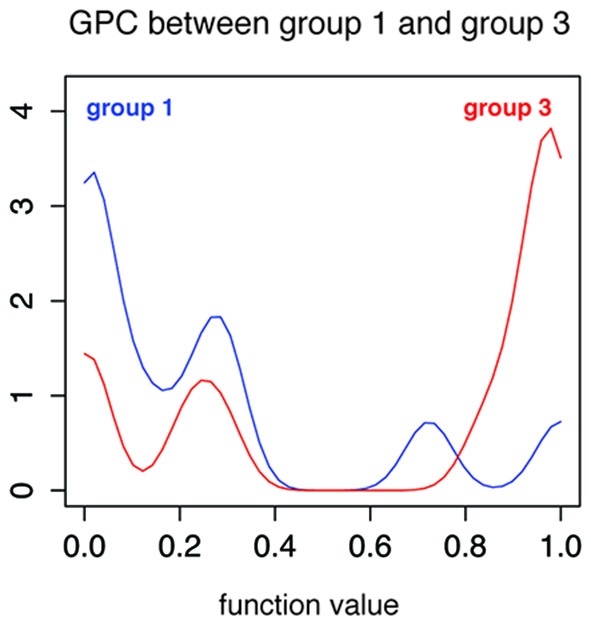
**Gaussian process classification between patient group 1 and patient group 3.** Histogram for the output of the Gaussian process function. Output values of less than 0.5 represent probability of belonging to group 1 (blue line) and values higher than 0.5 represent probability of belonging to group 3 (red line).

**Figure 4:**
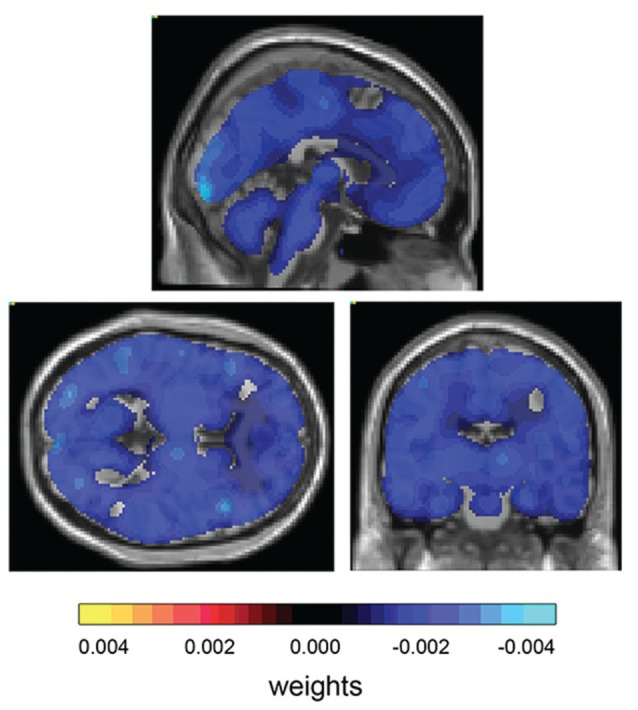
**Weight map for classification between patient group 1 and patient group 3.** Weight map for classification shows diffuse distribution of negative weights.

Due to high variability in neurological symptoms, we hypothesized that different patient groups would also present a distinct pattern of metabolic activity in their gray and white matter. To test whether de GPC algorithm could accurately classify the groups according to PET images, we performed three additional analyses comparing subsets of groups 1 and 2, groups 1 and 3 and groups 2 and 3. The results are presented in Table 3. Only group 1 and 3 could be accurately distinguished by the algorithm (accuracy of 72.7%, *p* = 0.026; Figure 3). These findings suggest a difference between the metabolic patterns of these two groups. The weight map presents a diffuse distribution of negative weights, suggesting a higher metabolism in group 3 when compared to group 1 (Figure 4).

**Table 3:** **Results for Gaussian processes classification between HTLV-1 patient groups**.

Class 1	NS	Sex	Age (SD)	Class 2	NS	Sex	Age (SD)	Acc.	*p*-value
Group 1	11	2M/9F	49.5 (10.7)	Group 2	11	2M/9F	49.2 (10.6)	68.2%	0.072
Group 1	11	4M/7F	49.9 (10.6)	Group 3	11	2M/9F	53.5 (11.8)	72.7%	0.026
Group 2	11	2M/9F	49.2 (10.6)	Group 3	11	3M/8F	51 (10)	54.5%	0.392

*Legend: NS = number of subjects, M = male, F = female, SD = Standard deviation, Acc = accuracy for GPC. Group 1 = EDSS 0, Group 2 = EDSS 1–2 and Group 3 = EDSS 2.5–10*.

## Discussion

In this study, we successfully applied GP classification to investigate patterns of metabolic activity in patients infected with HTLV-1 and healthy controls. Care was taken to ensure optimal matching for sex and age among compared groups, as well as including the same number of subjects in each group, in order not to bias the training of the classifier. The significance of our results was calculated using a 1000 fold random permutation of the input labels, to confirm that the classification was not due to a random association between classes and image features. This is particularly relevant in the analysis of small samples with a high number of features.

Our analysis shows that patients with HAM/TSP (group 3) could be classified with 85% accuracy (*p* = 0.003) when compared to controls. Furthermore, we found that asymptomatic HTLV-1 patients (group 1) could also be accurately classified when compared to the control group, with 85.7% accuracy (*p* = 0.001). These findings suggest that there is a distinct pattern of metabolic activity in HAM/TSP patients, differentiating them from the control group. Moreover, asymptomatic carriers’ brain metabolism also seems to differ from the control group, indicating changes in glucose uptake even in the absence of neurological symptoms. We also performed a multiclass GP classification between controls and patient groups 1 and 2, which could be classified with a mean accuracy of 69.7% (*p* = 0.001). The multiclass classification confirms that both patient groups have a distinct pattern of brain metabolism compared to controls (81.8% correct classification), but also from each other, with 72.7% correct classification of group 1 and 54.5% of group 3.

The generated weight maps show the joint contribution of all brain voxels in the class prediction. In the classification between asymptomatic HTLV+ (group 1) and controls, and between HAM/TSP (group 3) and controls, diffuse positive weights can be observed, which suggests a lower metabolism in the patient group compared to the control group. Classification of the group 2 (HTLV+ with few symptoms) showed a lower accuracy of 75% (*p* = 0.024). The generated map contained both negative and positive weights, with a diffuse distribution. These results might be related to the clinical heterogeneity of group 2, as it is composed of HTLV-1 patients with unspecific symptoms. If no clear and consistent pattern of metabolic change is present, the algorithm is unable to make accurate predictions during the LOOCV.

To further investigate the brain metabolism in our sample of HTLV-1 infected patients, we performed comparisons between the three patient groups. The only significant accuracy for classification was observed between asymptomatic HTLV+ (group 1) and HAM/TSP (group 3), but it was relatively small (72.7%, *p* = 0.026). The weight map suggests a diffuse lower metabolism in group 1 when compared to group 3.

Although inflammation plays a major role in the neuropathology of HTLV-1 infection (Bangham, 2000; Boxus and Willems, 2009; Olière et al., 2011; Lairmore et al., 2012) and usually causes an increase in 18F-FDG uptake, most of the glucose consumption in the brain is related to neuronal activity (Sokoloff, 1981). Therefore, the indirect measure of hypometabolism in HTLV-1 patients suggested by the weight map could be related to some degree of neuronal dysfunction caused by long-standing inflammation. Under this perspective, the predominantly positive weight map in the classification between patients and controls suggests some level of neuronal dysfunction, even in the asymptomatic group. These subjects did not present any neurological symptom as measured through the EDSS scale, although we found that asymptomatic HTLV-1 carriers already had changes in their neuropsychological performance (unpublished data).

Furthermore, there seems to be a distinction in the pattern of hypometabolism between these asymptomatic patients and the HAM/TSP group. Interestingly, the predominantly negative weight map suggests a lower metabolism in the asymptomatic group. One possible explanation for these results could be related to the mechanism of infection used by the virus. The Glucose Transporter Protein 1 (GLUT-1) is one of the identified proteins utilized by the HTLV-1 virus to infect cells (Jin et al., 2006; Jones et al., 2006; Kinet et al., 2007; Ghez et al., 2010). GLUT-1 is the primary glucose transporter across the BBB and is expressed in the luminal and abluminal membranes of endothelial cells (Simpson et al., 2007; Devraj et al., 2011). Considering the infection of these cells by the HTLV-1 virus using the GLUT-1 proteins (Afonso et al., 2008), we speculate that hypometabolism in the asymptomatic patients might be the result of direct impairment of glucose transport in the BBB through viral infection. In fact, it has been shown that the overexpression of HTLV-1 receptor binding domain in cell cultures altered glucose metabolism, which was consistent with the use of metabolite transporters as entry receptors by retroviruses (Sommerfelt, 1999; Ghez et al., 2010). However, this explanation is built over conjectures, and functional data is necessary to investigate the direct relationship between infection of endothelial cells and brain glucose metabolism, which might unveil interesting new aspects of the pathophysiology of HAM/TSP and related diseases.

Limitations of our study include the fact that, due to group matching, most of the analyses were performed with groups containing 10–11 subjects, which might limit the sensibility of the method. Furthermore, two of the subjects in group 3 were in use of corticosteroids, which might have affected the results. Nevertheless, we were able to show that asymptomatic HTLV-1 carriers have significant brain metabolic changes when compared to controls and that glucose uptake may be related to the pathophysiology of HTLV-1 infection. Further studies are necessary to confirm these results and shed more light on the relationship between infection of endothelial cells and brain glucose metabolism, which might lead to new therapeutic strategies.

## Author Contributions

MS contributed with the data analysis and interpretation of the data, drafting and critical revision of the manuscript. LCFR contributed with the design of the work, data acquisition, interpretation, drafting and critical revision of the manuscript. DVR contributed with data acquisition and critical revision of the manuscript. ABFC-P contributed with the design of the work, data acquisition and critical revision of the manuscript. RN contributed with data acquisition and critical revision of the manuscript. MAR-S contributed with the design of the work, interpretation of the data and critical revision of the manuscript. MB contributed with the data analysis and interpretation of the data and critical revision of the manuscript. DMM contributed with the interpretation of the data, design of the work, drafting and critical revision of the manuscript. All authors gave their approval on the final version of the manuscript and take accountability for all aspects of the work.

## Funding

This study was funded by Conselho Nacional de Desenvolvimento Científico e Tecnológico-CNPq (Grant Nos. 573646/2008-2 and 560035/2010-1) and Fundação de Amparo a Pesquisa de Minas Gerais-FAPEMIG (Grant No. CBB-APQ-000075-09). ABFC-P ABF is the recipient of a CNPq scholarship.

## Conflict of Interest Statement

The authors declare that the research was conducted in the absence of any commercial or financial relationships that could be construed as a potential conflict of interest.
